# Studying the correlation between different word sense disambiguation methods and summarization effectiveness in biomedical texts

**DOI:** 10.1186/1471-2105-12-355

**Published:** 2011-08-26

**Authors:** Laura Plaza, Antonio J Jimeno-Yepes, Alberto Díaz, Alan R Aronson

**Affiliations:** 1 Universidad Complutense de Madrid, Calle Profesor José García Santesmases s/n, 28040 Madrid, Spain; 2 National Library of Medicine, 8600 Rockville Pike, Bethesda, MD 20894, USA

## Abstract

**Background:**

Word sense disambiguation (WSD) attempts to solve lexical ambiguities by identifying the correct meaning of a word based on its context. WSD has been demonstrated to be an important step in knowledge-based approaches to automatic summarization. However, the correlation between the accuracy of the WSD methods and the summarization performance has never been studied.

**Results:**

We present three existing knowledge-based WSD approaches and a graph-based summarizer. Both the WSD approaches and the summarizer employ the Unified Medical Language System (UMLS) Metathesaurus as the knowledge source. We first evaluate WSD directly, by comparing the prediction of the WSD methods to two reference sets: the NLM WSD dataset and the MSH WSD collection. We next apply the different WSD methods as part of the summarizer, to map documents onto concepts in the UMLS Metathesaurus, and evaluate the summaries that are generated. The results obtained by the different methods in both evaluations are studied and compared.

**Conclusions:**

It has been found that the use of WSD techniques has a positive impact on the results of our graph-based summarizer, and that, when both the WSD and summarization tasks are assessed over large and homogeneous evaluation collections, there exists a correlation between the overall results of the WSD and summarization tasks. Furthermore, the best WSD algorithm in the first task tends to be also the best one in the second. However, we also found that the improvement achieved by the summarizer is not directly correlated with the WSD performance. The most likely reason is that the errors in disambiguation are not equally important but depend on the relative salience of the different concepts in the document to be summarized.

## Background

Word sense disambiguation (WSD) is an open problem of natural language processing (NLP) aimed at resolving lexical ambiguities by identifying the correct meaning of a word based on its context. A word is ambiguous when it has more than one sense (e.g. the word "cold", when used as a noun, may refer both to a respiratory disorder and to the absence of heat). It is the context in which the word is used that determines its correct meaning.

Word sense disambiguation is not an end in itself, but has obvious relationships with nearly every task that implies natural language understanding [1], such as text categorization [2], information extraction [3], machine translation [4,5] and sentiment analysis [6]. Focusing on the biomedical domain, the NLM (National Library of Medicine) Indexing Initiative [7] concluded that lexical ambiguity in the Unified Medical Language System (UMLS)^® ^was the biggest challenge in indexing biomedical journals with concepts from the UMLS Metathesaurus ^®^. Hunter and Cohen [8] argue that it is necessary to resolve the phenomenon of gene symbol polysemy in order to accurately recognize gene names in texts. Weeber et al. [9] found that, in order to replicate Swanson's literature-based discovery of the involvement of magnesium deficiency in migraine, it was important to resolve the ambiguity of an abbreviation *mg*, which can denote either "magnesium" or "milligram".

WSD has also been demonstrated to be an important step in knowledge-based approaches to automatic summarization [10]. When the UMLS, for instance, is used as knowledge source for biomedical summarization, the vocabulary of the document being summarized has to be mapped onto it. The majority of UMLS-based summarization systems rely on MetaMap [11] to translate the text into concepts from the UMLS Metathesaurus [12,13]. But, as stated by Shooshan et al. [14], the UMLS Metathesaurus contains a significant amount of ambiguity, and selecting the wrong mapping may bias the selection of salient information to sentences containing the wrong concepts, while discarding sentences containing the right ones.

To illustrate this assertion, consider the two following statements from a survey on the effect of vitamin C on the common cold:

s1* More evidence is needed before the conclusion that ascorbic acid has value in providing protection against the common cold*.

s2 *Vitamin C supplement to the diet may therefore be judged to give a "slight" advantage in reducing cold*.

Both sentences contain the ambiguous term "cold". MetaMap maps the phrase "common cold" in s1 to a single concept:

Meta Mapping (1000):

1000 C0009443:Cold (Common cold) [Disease or syndrome]

The confidence score, 1000, the Concept Unique Identifier or *CUI*, C0009443, and the UMLS semantic type for the concept, Disease or syndrome, are provided as output.

However, the phrase "in reducing cold" in s2 produces three different mappings where the term "cold" is assigned three possible UMLS Metathesaurus concepts, all of them with the same confidence score of 861:

Meta Mapping (888):

694 C0392756:Reducing (Reduced) [Qualitative concept]

861 C0234192:Cold (Cold sensation) [Physiologic function]

Meta Mapping (888):

694 C0392756:Reducing (Reduced) [Qualitative concept]

861 C0009264:Cold (Cold temperature) [Natural phen. or process]

Meta Mapping (888):

694 C0392756:Reducing (Reduced) [Qualitative concept]

861 C0009443:Cold (Common cold) [Disease or syndrome]

If the first of these mappings is chosen (C0234192 'Cold sensation'), the wrong meaning will be considered. A summarizer using MetaMap to map the text onto UMLS concepts would regard both sentences as talking about different topics. Even if the summarizer succeeds in identifying that the concept C0009443 'Common cold' is a central topic in the document, the sentence s2 could not be selected for the summary because it talks about a completely different meaning of "cold". Moreover, if the wrong mapping for "cold" is selected repeatedly throughout the document, the summarizer may even fail to determine that the concept 'Common cold' represents a salient topic in the document. Weeber et al. [15] estimated that around 11% of the phrases in MEDLINE ^® ^abstracts are mapped onto multiple CUIs and are hence ambiguous.

This paper presents the application of various WSD algorithms to improve biomedical summarization. We pursue two main goals. First, we want to test whether the results presented in [10], which showed that WSD improves the performance of a graph-based summarization system, may be extrapolated to more disambiguation algorithms. Second, we aim to find out if there is any correlation between the performance of the different WSD methods when evaluated directly (i.e., testing the disambiguation itself) and their contribution to the performance of the summarization task.

For the evaluation of WSD, we compare the prediction of the different methods to two reference sets. For the evaluation of the summarizer, we measure how the use of WSD affects the completion of a text summarization task. Both the WSD and summarization methods used in this work employ the UMLS Metathesaurus as the knowledge base. Our hypothesis is that using a WSD algorithm to choose between the candidate UMLS concepts returned by MetaMap will improve the performance of the summarizer, and that the more accurate the WSD algorithm, the greater the improvement obtained in summarization. The paper is organized as follows. We first introduce the UMLS and the MetaMap program and present some related work in biomedical WSD and summarization. Then, we describe three methods for WSD and a graph-based summarization system. We next present the evaluation methodology, and later discuss the results of the evaluation of the various WSD methods and the summarizer. The final section provides concluding remarks.

### Related work

In this section, we introduce the UMLS and the MetaMap program and present some previous work in biomedical WSD and automatic summarization.

### UMLS and MetaMap

The NLM's UMLS (Unified Medical Language System) [16,17] provides a large resource of knowledge and tools to create, process, retrieve, integrate and/or aggregate biomedical and health data. The UMLS has three main components:

• Metathesaurus, a compendium of biomedical and health content terminological resources under a common representation, which contains lexical items for each one of the over 1 million concepts and relations among them.

• Semantic Network, which provides a categorization of Metathesaurus concepts into the 133 currently available semantic types. In addition, it includes relations among semantic types.

• SPECIALIST Lexicon, containing lexical information for over 200,000 terms required for natural language processing which covers commonly occurring English words and biomedical vocabulary.

Concepts in the Metathesaurus denote possible senses that a term may have. They are assigned a unique identifier (CUI) which has linked to it a set of terms which denote alternative ways to represent the concept, for instance, in text. These terms, depending on the availability, are represented in several languages. Only English terms are used in this work. Concepts are assigned one or more semantic types. Concepts may have a definition linked to them and sometimes more than one from multiple sources. Relations between concepts are often available. All the information about a concept can be traced back to the resource from which it was collected.

The **MetaMap **program [11] maps biomedical text to concepts in the UMLS Metathesaurus. The semantic type for each concept mapping is also returned. MetaMap employs a knowledge intensive approach which uses the SPECIALIST Lexicon in combination with lexical and syntactic analysis to identify noun phrases in text. The mappings between a noun phrase and one or more Metathesaurus concepts is computed by generating lexical variations and allowing partial matches between the phrase and concept. The possible UMLS concepts are assigned scores based on the closeness of match of the input noun phrase to the target concepts.

### WSD of Biomedical Text

The most popular approaches to WSD in the biomedical domain are based on supervised learning [18-20], which has shown better performance than alternative approaches (see Schuemie et al. [21] for a review of WSD methods in the biomedical domain). Supervised WSD aims to train classifiers that assign senses to words in text using machine learning techniques.

Supervised WSD embodies the main disadvantage of needing manually annotated data which are often unavailable and may be impractical to create. An alternative that has been shown to achieve interesting results is unsupervised WSD methods. These approaches exploit the information in existing resources like a lexical knowledge base to perform disambiguation, without using annotated data. In particular, in the biomedical domain, different domain-independent approaches have been adapted to employ the UMLS as the knowledge base, and thus used to disambiguate biomedical text.

Among the unsupervised WSD methods we find journal descriptor indexing (JDI) [22], disambiguation based on concept profiles [23], disambiguation based on context examples collected automatically [24] and graph-based approaches [25].

### Summarization of Biomedical Text

Extractive text summarization can be defined as the process of determining salient sentences in a text. These sentences are expected to condense the relevant information regarding the main topic covered in the text. We focus on graph-based summarization methods, since the summarizer used in this work falls into this category of techniques (see [26] for a more thorough study of domain-independent summarization techniques and [27] for biomedical-focused approaches).

Graph-based summarization methods have recently attracted much attention from the research community [28-30]. They usually represent the documents as graphs, where the nodes correspond to text units such as words, phrases or sentences, and the edges represent cohesion or similarity relations between these units. Once the document graph is created, salient nodes within it are discovered and used to extract the corresponding units for the summary.

In the biomedical domain, the UMLS has proved to be a useful knowledge source for summarization [12,13,30]. For example, Reeve et al. [13] adapt the lexical chaining approach [31] to use UMLS concepts rather than terms, and apply it to single document summarization. To discover the UMLS concepts within the text, the MetaMap Transfer Tool is used. BioSquash [32] is a question-oriented extractive system for biomedical multi-document summarization. It constructs a graph that contains concepts of three types: ontological concepts (general ones from WordNet [33] and specific ones from the UMLS), named entities and noun phrases. A more complex work is presented in Fiszman et al. [12]. They propose an abstractive approach that relies on the semantic predications provided by SemRep [34] to interpret biomedical text, and on a transformation step using lexical and semantic information from the UMLS, to produce abstracts from biomedical scientific articles.

## Methods

In this section, we first present three unsupervised WSD methods: the Journal Descriptor Indexing (JDI), the Machine Readable Dictionary (MRD) and the Automatic Extracted Corpus (AEC). The description of these methods here is a summary of the description available in [24]. We next describe a summarization algorithm that uses the UMLS concepts and relations to construct a graph-based representation for the document to be summarized.

### WSD Methods

#### Journal Descriptor Indexing (JDI)

The Journal Descriptor Indexing (JDI) method developed by Humphrey et al. [22] is a well-known unsupervised technique for WSD in the biomedical domain. JDI uses the semantic types assigned to Metathesaurus concepts to perform disambiguation. Journal Descriptors (JD) are general MeSH^® ^headings assigned to the journals in MEDLINE. The JDI technique assigns a score to the semantic types which allows selecting the highest ranking semantic type of the target ambiguous word. The selected semantic type is used to identify the proper concept in the Metathesaurus under the assumption that each ambiguous word is assigned to a distinct semantic type. The score is estimated by comparing the JD indexing of the context of the ambiguous word and the pre-calculated JD indexing of the semantic types using cosine similarity. This score represents the confidence in the indexing of the JD. JD indexing relies on building, for each JD, a vector of words based on a training data of citations extracted from MEDLINE. Words in the vector are assigned probabilities estimated by counting the number of times a word is related to a JD divided by the total number of citations. Pre-calculated JD indexing of the semantic types is built for each semantic type comparing a word vector of semantic types to the JD word vectors. Words are extracted from the concepts in the Metathesaurus assigned to each semantic type. Detailed examples of use are available in [22]. This approach, however, has the limitation of not being able to disambiguate cases where the concepts linked to the ambiguous word are assigned the same semantic type. For instance, two concepts of the word *frequency *(*spatial frequency *with CUI C0871396 and *statistical frequency *with CUI C1705502) belong to the semantic type *Quantitative Concept*.

#### Machine Readable Dictionary (MRD)

This knowledge-based WSD method compares the context of the ambiguous word to the information available in a knowledge source about each of the candidate senses. This algorithm can be seen as a relaxation of Lesk's algorithm [35], which relies on the matches of words in the sense definitions overlapping with words in the definition of neighboring words. The algorithm is very expensive since the sense combination might be exponentially large even for a single sentence. Vasilescu et al. [36] have shown that similar or even better performance might be obtained disambiguating each ambiguous word separately. We follow this approach and, for each candidate concept of an ambiguous word, a profile is built and then compared to the context of the ambiguous word, selecting the concept with the highest matching score. This approach has been previously used by McInnes [23] in the biomedical domain with the NLM WSD corpus.

For each candidate concept, a profile is generated. The concept profile is represented in a vector space in which each dimension is one of the unique words in the profile. The words from the concept profile are obtained from the concept definition or definitions, if available, synonyms, and related concepts excluding siblings, from the UMLS. Stop words are discarded, and Porter stemming is used to normalize the words. In addition, the word frequency is normalized based on the *inverted concept frequency *so that terms which are repeated many times within the UMLS will have less relevance.

To compare the context of the ambiguous word to the concept profiles, the context is turned as well into a vector representation. This context vector includes the word frequency in the context. Stop words are removed and the Porter stemmer is used to normalize the words.

In this machine readable dictionary approach (MRD), concept profiles *C_w _*linked to an ambiguous word *w *and word context *cx *are compared using cosine similarity as shown in equation 1. The concept *c *in *C_w _*with the highest cosine similarity is selected.

(1) $$MRD\left(c\right)=\underset{c\in C_{w}}{\text{argmax}}\frac{c\cdot cx}{\left|c||cx\right|}$$

#### Automatic Extracted Corpus (AEC)

WSD approaches based on supervised learning require training data which is expensive to obtain by manual annotation. To overcome this problem, corpora to train statistical learning algorithms for ambiguous terms can be automatically obtained by retrieving documents from a large corpus. Queries are generated using English *monosemous relatives *[37] of the candidate concepts available from the knowledge source. These terms are used to collect examples of the context in which the ambiguous word is used which can be used to train a supervised learning algorithm.

In our work, the list of candidate relatives includes synonyms and terms from related concepts obtained from the UMLS. We consider a term as monosemous if it is only assigned to one concept in the Metathesaurus. This means that *cold *is ambiguous since it is linked to more than one concept in the Metathesaurus, while *cold storage *is monosemous because it is only linked to the concept with CUI *C0010405*.

We have used EUtils [38] from PubMed^® ^[39] as the search engine to retrieve documents from MEDLINE. The query language used by PubMed is based on Boolean operators and allows for field searches, e.g., it allows searching a specific term within the metadata. Monosemous synonyms are added to the query and joined with the OR operator. Monosemous terms from related concepts are combined with the AND operator and the ambiguous term assuming one sense per collocation, then combined with monosemous synonyms using the OR operator. In order to retrieve documents where the text (title or abstract of the citation) contains the query terms, the *[tiab] *search field is used. Quotes are used to find exact mentions of the terms in phrases and increase precision.

**Figure 1:**
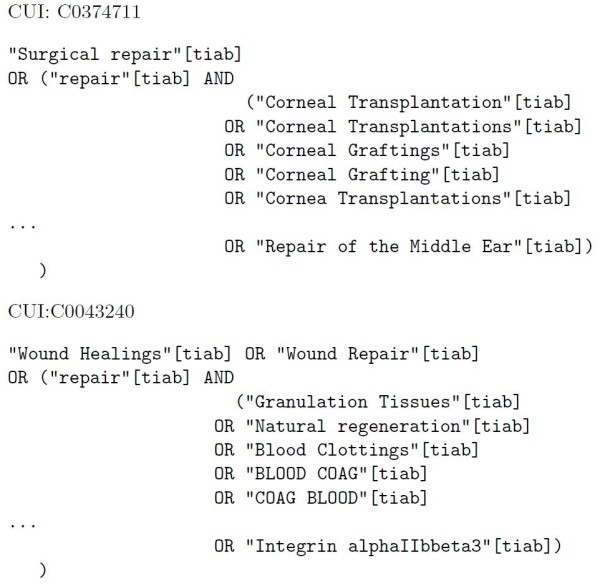
**Example query for term repair used in the AEC method**. This example shows the two queries generated for each one of the candidate senses of the term *repair*. The first sense, with CUI *C0374711*, is related to surgical repair while the second sense, with CUI *C0043240*, is related to wound healing.

Since the terms are used in a retrieval system, we have performed further filtering to the selected monosemous terms. Long terms (more than 50 characters) are not considered since these terms are unlikely to appear in MEDLINE. This prevents having unnecessarily long queries which could be problematic with retrieval systems. Very short terms (less than 3 characters) and numbers are not considered to avoid almost certain ambiguity. A standard stop word list is used to remove uninformative English terms. Figure 1 shows example queries for each sense of the term *repair*. The first query is related to the UMLS concept *surgical repair *(CUI: C0374711, a human performed activity) while the second is related to *wound healing *or *tissue repair *(CUI: C0043240, a biological funcion). As we can see in the first section of the query in Figure 1, we find the monosemous synonyms of the term repair for each of the senses. Then, we find the ambiguous word (*repair *in this example) which is combined with related monosemous terms, which we assume appear only with that candidate sense of the ambiguous word.

Documents retrieved using PubMed are assigned to the concept which was used to generate the query. If no documents are returned for a given query, the quotes are replaced by parentheses to allow finding the terms in any position in the title or abstract text. This corpus is used to train a statistical learning algorithm. In this work we have used Naïve Bayes. Disambiguation is performed using the trained model with new disambiguation examples.

We have evaluated several limits on the number of retrieved documents. Since there is not a significant difference in performance, 100 documents are collected from MEDLINE for each concept identifier.

### Summarization Method

We use the graph-based summarization method presented in [10], which we briefly explain here for completeness (see [10] for additional details). The method consists of the following 4 main steps:

• The first step, **concept identification**, is to map the document to concepts from the UMLS Metathesaurus and semantic types from the UMLS Semantic Network. We first run the MetaMap program over the text in the body section of the document. MetaMap returns the list of candidate mappings, along with their score. To accurately select the correct mapping when MetaMap is unable to return a single best-scoring mapping for a phrase, we use the various WSD methods explained in the previous section. UMLS concepts belonging to very general semantic types are discarded since they have been found to be excessively broad and do not contribute to summarization. These types are *Quantitative concept*, *Qualitative concept*, *Temporal concept*, *Functional concept*, *Idea or concept*, *Intellectual product*, *Mental process*, *Spatial concept *and *Language*.

• The second step, **document representation**, is to construct a graph-based representation of the document. To do this, we first extend the disambiguated UMLS concepts with their complete hierarchy of hypernyms (*is a *relations) and merge the hierarchies of all the concepts in the same sentence to construct a *sentence graph*. The two upper levels of these hierarchies are removed, since they represent concepts with excessively broad meanings. Next, all the sentence graphs are merged into a single *document graph*. This graph is extended with two further relations (*other related *from the Metathesaurus and *associated with *from the Semantic Network) to obtain a more complete representation of the document. Finally, each edge is assigned a weight in [0, 1] as shown in equation 2. The weight of an edge *e *representing an *is a *relation between two vertices, *v_i _*and *v_j _*(where *v_i _*is a parent of *v_j_*), is calculated as the ratio of the depth of *v_i _*to the depth of *v_j _*from the root of their hierarchy. The weight of an edge representing any other relation (i.e., *associated with *and *other related*) between pairs of leaf vertices is always 1.

(2) $$\begin{array}{c} weight\left(e,v_{i},v_{j}\right)=\beta \\ where\left\{\begin{array}{cc} \beta =\frac{depth\left(v_{i}\right)}{depth\left(v_{j}\right)} & \text{if}\;e\;\text{representsan}\;is\text{\_}a\;\text{relation}\\ \beta =1 & \text{otherwise} \end{array}\right) \end{array}$$

• The third step, **topic recognition**, consists of clustering the UMLS concepts in the document graph using a degree-based clustering method similar to PageRank [40]. The aim is to construct sets of concepts strongly related in meaning, based on the assumption that each of these clusters represents a different topic in the document. We first compute the *salience *or *prestige *of each vertex in the graph, as the sum of the weights of the edges that are linked to it. Next, the nodes are ranked according to its salience. The *n *vertices with the highest salience are labeled as *hub vertices*. The clustering algorithm then groups the hub vertices into *hub vertex sets *(HVS). These can be interpreted as sets of concepts strongly connected and will represent the centroids of the final clusters. The remaining vertices (i.e., those not included in the HVS) are iteratively assigned to the cluster to which they are most connected. The output of this step is, therefore, a number of clusters of UMLS concepts, with each cluster represented by the set of most highly connected concepts within it (the so-called HVS).

• The last step, **sentence selection **consists of computing the similarity between each sentence graph (*S_i_*) and each cluster (*C_i_*), and selecting the sentences for the summary based on these similarities. To compute sentence-to-cluster similarity, we use a non-democratic vote mechanism [41] so that each vertex of a sentence assigns a vote to a cluster if the vertex belongs to its HVS, half a vote if the vertex belongs to it but not to its HVS, and no votes otherwise. The similarity between the sentence graph and the cluster is computed as the sum of the votes assigned by all the vertices in the sentence graph to the cluster. Finally, a single score for each sentence is calculated, as the sum of its similarity to each cluster adjusted to the cluster's size (equation 3). The *N *sentences with highest scores are then selected for the summary.

(3) $$Score\left(S_{j}\right)=\underset{c_{i}}{\sum }\frac{similarity\left(C_{i},S_{j}\right)}{\left|C_{i}\right|}$$

### Evaluation Methods

This section describes the methodology followed to evaluate both the WSD and the summarization performance when the different WSD algorithms are used for concept identification.

#### WSD evaluation

The evaluation of disambiguation is performed by comparing a reference set with the prediction of the WSD methods. Evaluation of WSD methods is presented in terms of accuracy, defined in equation 4, where an instance is an example of an ambiguous word to disambiguate.

(4) $$Accuracy=\frac{Instances\;Correctly\;Predicted}{All\;Instances}$$

The JDI approach, as mentioned above, cannot deal with cases where candidate concepts have the same semantic type. Therefore, for each experiment, we compute the average accuracy over the whole set and the JDI subset, where the ambiguous terms having candidate mappings from the same semantic type have been removed. Moreover, we evaluate each WSD algorithm over two different datasets:

• The **NLM WSD dataset **[15,42] contains 50 ambiguous terms which have been annotated with a sense number. Each sense number has been related to UMLS semantic types. 100 manually disambiguated cases are provided for each term. In case no UMLS concept is appropriate, *None of the above *has been assigned in the NLM WSD.

The selection of the 50 ambiguous words was based on an ambiguity study of 409,337 citations added to MEDLINE in 1998. MetaMap was used to annotate UMLS concepts in the titles and abstracts based on the 1999 version of the UMLS. Out of 4,051,445 ambiguous cases found in these citations, 552,153 cases are represented by these 50 terms. This means that a large number of ambiguous cases can be solved disambiguating these highly frequent terms. A team of 11 people annotated the ambiguous cases with Metathesaurus entries.

The dataset is available from [43]. In addition, from the same site [42] it is possible to obtain the version of the UMLS used for the development of the NLM WSD dataset which we have used in our work. Recently, a mapping to the 2007AB version of the Metathesaurus has been made available.

For the evaluation, we have considered the same setup as Humphrey et al. [22] and discarded the *None of the above *category. Since the ambiguous term *association *has been assigned entirely to *None of the above*, it has been discarded. This means that we will present results for 49 out of the 50 ambiguous terms.

• The **MSH WSD dataset **was developed automatically using MeSH indexing from MEDLINE [44]. This automatically developed set is based on the 2009AB version of the Metathesaurus and MEDLINE up to May 2010 using PubMed to recover the documents. The Metathesaurus is screened to identify ambiguous terms which contain MeSH headings. Then, each ambiguous term and the MeSH headings linked to it are used to recover MEDLINE citations using PubMed where the term and only one of the MeSH headings co-occur. The term found in the MEDLINE citation is assigned the UMLS concept identifier linked to the MeSH heading. Because this initial set is noisy, we have filtered out some of the ambiguous terms to enhance precision of the set. The filtering process targeted cases where at least 15 examples are available for each sense, filtered out noisy examples and ensured that each ambiguous word has more than one character. This filtered set has 203 ambiguous terms and includes not only words but abbreviations which, in some cases, are used as terms. In addition, it covers a larger set of semantic types compared to the NLM WSD set. The MSH WSD set is broken into three sections: Abbreviation Set, Term Set and the Term/Abbreviation Set. The Abbreviation Set contains 106 ambiguous acronyms. The Term set contains 88 ambiguous terms, and the Term/Abbreviation Set contains 9 ambiguous term/abbreviations.

#### Summarization evaluation

The most common approach to evaluating automatically generated summaries of a document (also known as *peers*) is to compare them against manually-created summaries (*reference *or *model *summaries) and measure the similarity between their content. The more content that is shared between the peer and reference summaries, the better the peer summary is assumed to be.

In this work, the ROUGE metrics [45,46] are used to quantify the content similarity between the automatic summaries and the reference ones. ROUGE is a commonly used evaluation method for summarization which uses the proportion of n-grams between a peer and one or more reference summaries to compute a value within [0, 1] which estimates the content that is shared between them. The following ROUGE metrics are used: ROUGE-2 and ROUGE-SU4. ROUGE-2 counts the number of bigrams that are shared by the peer and reference summaries and computes a recall-related measure as follows [46]:

(5) $$\frac{\sum_{S\in \left\{Reference\;Summaries\right\}}\sum_{bigram\in S}Count_{match}\left(bigram\right)}{\sum_{S\in \left\{Reference\;Summaries\right\}}\sum_{bigram\in S}Count\left(bigram\right)}$$

where *Count_match_*(*bigram*) is the maximum number of bigrams co-occurring in a candidate summary and a set of reference summaries. Similarly, ROUGE-SU4 measures the overlap of skip-bigrams (i.e., pairs of words in their sentence order, allowing for arbitrary gaps) between the peer and reference summaries, using a skip distance of 4. Both ROUGE-2 and ROUGE-SU4 have shown high correlation with the human judges gathered from the Document Understanding Conferences [47].

As the evaluation corpus, we use a collection of 150 full-text scientific articles randomly selected from the BioMed Central corpus for text mining research [48]. As stated in [46], this collection is large enough to ensure significant results in the ROUGE evaluation.

In order to quantitatively evaluate the impact of WSD on biomedical text summarization, we measure the improvement achieved in the summarization performance by applying the different WSD methods for mapping documents onto concepts in the UMLS Metathesaurus. The 2007AC version of the UMLS is used. We generate automatic summaries by selecting sentences until the summary is 30% of the original document size, and use the abstract of the papers (i.e., the authors' summaries) as reference summaries. The abstracts of scientific articles have been frequently used as gold standards for summarization evaluation [13], as well as to automatically and semiautomatically produce such gold standards [49-51].

## Results and Discussion

In this section, we analyze and discuss the results obtained in the evaluation. First, we present the results of the WSD itself. Second, we show the ROUGE scores for the summaries generated using the various WSD algorithms for identifying UMLS Metathesaurus concepts. Finally, we compare the WSD and summarization results in order to find out if there exists any correlation in the behavior of the different disambiguation methods.

### WSD Results

Direct evaluation of the WSD methods is provided for two available datasets: the NLM WSD and the MSH WSD.

Table 1 shows the accuracy obtained by the disambiguation methods in the NLM WSD set. These results are a summary of the results available in [24]. We find that the JDI method achieves better performance compared to other knowledge-based methods with the subset, 44 out of the 49 ambiguous words. It must be noted that the JDI algorithm performs particularly well with the NLM WSD subset, where all candidate senses of the ambiguous terms are assigned different semantic types, so that JDI is able to distinguish between possible senses.

**Table 1:** NLM WSD results: method comparison

WSD Method	Set	Subset
MRD	0.6389	0.6526
AEC	0.6836	0.6932
JDI		**0.7475**

MRD stands for Machine Readable Dictionary, AEC stands for Automatic Extracted Corpus and JDI stands for Journal Descriptor Indexing. The term *set *stands for all the ambiguous words in the NLM WSD test set while *subset *indicates that only the words usable by the JDI method are considered. This means, the words with different semantic types.

The MRD approach produces results which are not as good as the AEC results. There are several possible explanations for this. MRD relies on the terms presented in the dictionary, in this case the UMLS Metathesaurus. We identify related terms, but in some cases these terms are not representative of the context for a given sense. We also realize that the AEC queries are not specific enough in some cases, so they retrieve false positives for a given sense. The results are in keeping with general English results, where the performance is lower than using manually generated training data.

There are some terms which are difficult to disambiguate because the senses are very close in meaning. For instance, the term *blood pressure *in the Metathesaurus could indicate the *blood pressure determination *procedure or the *blood pressure level *of a patient. These senses are difficult to distinguish and the UMLS did not provide enough information to generate a query for each concept denoting *blood pressure *which would allow retrieving distinctive citations to train a classifier to perform WSD.

Table 2 shows the overall accuracy of the disambiguation methods using the MSH WSD set. Since the JDI method is only able to disambiguate ambiguous terms or abbreviations whose candidate senses do not share the same semantic type. There exist 44 ambiguous terms in which this method is not able to distinguish between the possible senses.

**Table 2:** MSH WSD results: method comparison

Dataset	AEC	JDI	MRD
Abbreviation Set	**0.9090**		0.8759
Abbreviation Subset	**0.9218**	0.6725	0.8838

Term Set	**0.7462**		0.7148
Term Subset	**0.7448**	0.6209	0.7132

Term/Abbreviation Set	**0.8879**		0.8801
Term/Abbreviation Subset	**0.9026**	0.6899	0.8715

Overall Set	**0.8383**		0.8070
Overall Subset	**0.8448**	0.6551	0.8118

AEC stands for Automatic Extracted Corpus, MRD stands for Machine Readable dictionary, and JDI stands for Journal Descriptor Indexing. The term *set *stands for all the ambiguous words in the category while *subset *indicates that only the words usable by the JDI method are considered.

Considering the three subsets which comprise the MSH WSD dataset, the Term Set is most difficult to disambiguate. This indicates that the contextual difference between ambiguous terms is more finely grained than the contextual differences between abbreviations.

Generally, all of the methods obtain a higher accuracy in disambiguating ambiguous terms from the Abbreviations set than from the Term set. Since the long form of the abbreviation might be present in many cases, this itself could provide enough context for the algorithms to disambiguate between them. The AEC method obtains the best accuracy on the MSH WSD dataset. It should be noted that AEC relies on the UMLS content to collect documents from MEDLINE which might expand the context terms and, in addition, relies on statistical learning approaches which might produce a better partition of the feature space.

In contrast, the JDI method obtains the lowest disambiguation accuracy of the methods used with the MSH WSD set. This is surprising compared to the results obtained with the NLM WSD set. The reasons for this behavior are mainly related to the granularity of JDs used to index the semantic types, and the context of the ambiguous word. The NLM WSD set contains a smaller number of semantic type combinations which seem to perform reasonably well; but in the MSH WSD set, the combination is larger and combines semantic types with a smaller number of sample terms in the Metathesaurus. The MSH WSD offers a wider coverage of the biomedical domain. The JDI method performs JD indexing on the context of the ambiguous words. Sometimes the context might not provide enough evidence to produce a reasonable indexing. Another issue might be related to the indexing of semantic types with JDs. We have observed that some indexing of semantic types with JDs assign top ranking to a set of JDs which do not look as relevant to the category (i.e., *Temporal concept *or *Intellectual property*). This might mean that, for some semantic types, either there are no appropriate JDs to index them or the terms collected as features from the Metathesaurus do not provide enough evidence for a proper indexing.

In this dataset, MRD behaves better than JDI, but not as well as AEC. There are some cases in which the MRD approach cannot disambiguate ambiguous terms properly. Some of these terms might be confusing in context (e.g., *man*), so that, in these cases, the concept profiles might not be representative of the ambiguous term senses. So, the terms with higher *tf *× *idf *are not representative of the context of the ambiguous words. Besides, we have observed that for some MRD cases, the context surrounding the senses for each of these ambiguous words is not distinct enough to accurately disambiguate between them.

### Summarization Results

We next present the evaluation results for the summarization task. The ROUGE scores for the summaries generated using different word sense disambiguation algorithms (JDI, MRD and AEC) are shown in table 3. We also show the ROUGE scores for the summaries generated by selecting the first CUI returned by MetaMap ("First Mapping"). Since the order of equally scored concepts returned by MetaMap is not informative, this strategy is essentially the same as not using WSD at all. Consequently, we consider this approach to be a baseline method against which others are compared.

**Table 3:** ROUGE scores for the summaries generated using different WSD strategies

Summarizer	ROUGE-2	ROUGE-SU4
AEC	**0.3670**	**0.3379**
MRD	0.3611	0.3341
JDI	0.3538	0.3267
First mapping	0.3283	0.3117

MRD stands for Machine Readable Dictionary, AEC stands for Automatic Extracted Corpus and JDI stands for Journal Descriptor Indexing. Systems are sorted by decreasing ROUGE-2 score.

The results in table 3 show that, regardless of the method employed, using word sense disambiguation improves the average ROUGE scores for the summarizer when compared against the "First mapping" baseline. According to a pairwise Wilcoxon Signed Ranked Test performed with Bonferroni correction for multiple testing (*p <*0.01), all disambiguation algorithms significantly improve ROUGE-2 and ROUGE-SU4 metrics, compared with no WSD (i.e., "First mapping").

Regarding comparison among different WSD algorithms, the best results are obtained using AEC, followed by MRD and, finally, JDI. Table 4 shows significance values for each pair of algorithms. We observe that MRD and AEC produce equivalent summarization results, while both methods significantly improve ROUGE scores compared with JDI.

**Table 4:** p values for statistical significance (Wilcoxon Signed Ranks Test)

Summarizer	ROUGE-2	ROUGE-SU4
MRD-AEC	0.187	0.341
JDI-AEC	0.013	0.058
JDI-MRD	0.057	0.084

MRD stands for Machine Readable Dictionary, AEC stands for Automatic Extracted Corpus and JDI stands for Journal Descriptor Indexing.

On the other hand, a careful analysis of the ROUGE-2 scores obtained for each document in the evaluation corpus when the different WSD algorithms are used has shown no clear dominance of a single algorithm. However, it has been found that, on average, AEC and MRD behave better than JDI for most documents. Specifically, AEC achieves the highest ROUGE-2 scores for 62 documents, MRD for 52 and JDI for 32. However, AEC behaves better than JDI in 94 documents, and better than MRD in 84 documents. In turn, MRD behaves better than JDI in 88 documents. Similar results are obtained for the ROUGE-SU4. According to this metric, the summaries generated using AEC are better than those obtained using JDI for 90 documents, and better than those of MRD for 77 documents. Concerning MRD, it obtains better ROUGE-SU4 scores than JDI in 91 documents.

## Discussion

We first discuss the results of the summarization evaluation in relation to those presented in [10]. The main conclusion coincides: applying WSD to the output of MetaMap as part of the graph-based summarizer improves the quality of the summaries that are generated. However, Plaza et al. [10] reported an improvement of ≈ 7.5% in ROUGE-2 and ≈ 4.7% in ROUGE-SU4 for the best WSD algorithm, i.e., Personalized PageRank word-to-word (*ppr-w2w*) [25]. In the present work, the improvement achieved by the best disambiguation method (AEC) is ≈ 12% in ROUGE-2 and ≈ 8.4% in ROUGE-SU4. Moreover, even the worst performance method (JDI) improves the summarization results achieved in [10].

**Figure 2:**
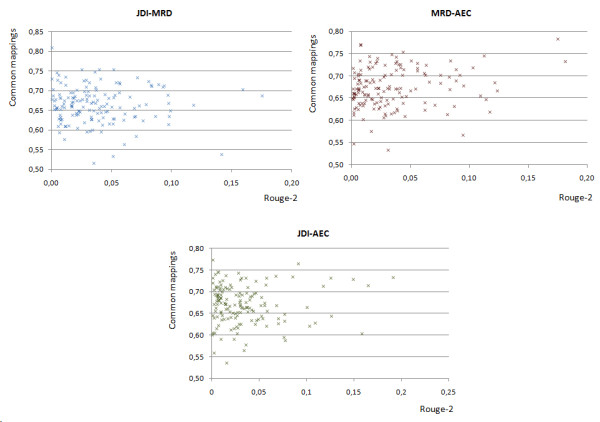
**Comparison between the disambiguation performed by different WSD algorithms and the ROUGE-2 scores obtained by the summaries generated using each WSD algorithm**. Disambiguation performance is represented in terms of the proportion of common mappings between each pair of WSD algorithms, while their performance in the summarization task is pictured in terms of the difference in the ROUGE-2 scores achieved by the summaries generated. Each data point in these graphs represent a different document from the evaluation corpus. MRD stands for Machine Readable Dictionary, AEC stands for Automatic Extracted Corpus and JDI stands for Journal Descriptor Indexing.

We next compare the summarization results to those of the direct WSD evaluation. We find that summarization results are consistent with those obtained when evaluating the various WSD algorithms on the MSH WSD dataset. In both evaluations, the worst performing method is JDI. MRD significantly improves upon the results of the JDI algorithm, while AEC slightly improves upon MRD. However, the results differ from the NLM WSD dataset, where the JDI approach achieves the best results. The reason for this finding was previously discussed in the section devoted to the WSD evaluation, when comparing the disambiguation results for both evaluation datasets. As with the MSH WSD dataset, the summarization corpus presents a large number of semantic type combinations that explain the poor accuracy of JDI. Moreover, the contexts of the ambiguous terms in the summarization corpus are expected to present similar characteristics to those in the MSH WSD corpus, which was generated automatically. In order to better understand the relationship between the WSD and the summarization results, we compare, for each document in the summarization corpus, the disambiguation produced by each pair of WSD algorithms (in terms of the proportion of "common mappings"), with their performance in the summarization task when they are used to map the text onto UMLS Metathesaurus concepts (in terms of the difference in the ROUGE-2 scores they achieve). A "common mapping" is a mapping where MetaMap returns various candidate CUIs for the same phrase (see the sentence s2 in the Introduction section) and the two WSD algorithms produce the same disambiguation decision (i.e., they select the same candidate concept). Our hypothesis is that the higher the percentage of agreement in the disambiguation produced by the two algorithms, the more similar the document graphs they produce in the summarization method, and thus the more similar the summaries that are generated. Consequently, when two WSD algorithms produce similar disambiguation decisions, the ROUGE values for the corresponding automatic summaries are expected to be close. These results are shown in Figure 2, where each chart represents the comparison among a pair of WSD algorithms.

However, we observe no linear relation between the two variables, i.e., as the percentage of agreement in the concept mappings produced by the WSD methods increases, the difference in their ROUGE-2 scores does not decrease in the same proportion. In contrast, for every pair of WSD algorithms, we found documents where a high agreement in the disambiguation is achieved, but the disagreement in the ROUGE-2 values is also relatively high and conversely, documents with low disagreement in the disambiguation, but high agreement in the ROUGE-2 values.

To clarify the reasons for this finding, we carefully examined, for each pair of WSD algorithms, the two extreme documents (that is, the document with the highest agreement of concept mappings and the highest disagreement of ROUGE-2 scores, and the document with the highest disagreement of concept mappings and the highest agreement of ROUGE-2 scores). We hypothesize the following conclusions that may explain the non-linear relation between the WSD and the summarization results:

• Even when two algorithms agree in a great number of concept mappings, we do not know the accuracy of such mappings. Moreover, when two algorithms produce different mappings for a given concept, it may mean that (1) neither of them has produced the correct mapping or (2) only one of them has produced the correct mapping. Therefore, depending on the accuracy of each WSD algorithm in the non-common mappings, the result of the summarization process may vary.

• The summarizer is not equally sensitive to all errors in disambiguation. Note that it is more important to assign the correct meaning to the concepts that represent the topic of the document, especially to those belonging to the HVS or centroids of the cluster that are generated by the summarization method since these concepts have more influence on the final selection of sentences for the summaries. For instance, we found a document about the role of survivin in embryonic submandibular gland development, where the phrase *submandibular gland *is mapped to three different concepts by AEC, MRD and JDI (C1522654, C0038556 and C1268976, respectively). Since these concepts act as HVS in the clustering process, they greatly influence the final selection of sentences for the summary. As a result, even if, on average, the disambiguation produced by the three algorithms is quite similar, the resulting summaries are significantly different. In contrast, it has been found that, for those documents with a high agreement in the WSD results, if there is agreement in the mapping assigned to the central concepts in the document, then the ROUGE-2 scores obtained are also relatively similar.

• An important percentage of the discrepancy in the WSD mappings for the documents that have been analyzed correspond to concepts belonging to those semantic types considered as "too generic" by the summarization method. Note that such concepts are discarded and not taken into account in the summarization process. Examples of concepts from generic semantic types where the various WSD methods disagree are *change*, *identify *and *stage*.

• It must be observed that, when the wrong mapping is selected for a concept, the summarization method extends the document graph with incorrect hypernyms and relations. This occurs, for instance, in the document previously mentioned, where the term *p53 *is mapped to the concept *Protein p53 (C0080055) *by AEC, and to the concept *TP53 wt Allele (C1705526) *by MRD. When both concepts are extended with their hypernyms, the resulting hierarchies are *[Protein, Organized by Function (C0815043) - Regulatory Protein (C0815047) - Cell Cycle Protein (C0243021) - Protein p53 (C0080055)] *and *[TP53 Gene (C0079419) - TP53 wt Allele (C1705526)]*, respectively. Note that the hierarchies are significantly different, the final concepts even belonging to different semantic types (*Amino Acid, Peptide, or Protein (T116) *versus *Gene or Genome (T028)*). Therefore, it cannot be expected that the relationship between the disambiguation and the summarization results is linear.

• We think that the summarizer itself is implicitly performing some sort of WSD, which benefits from information from both the Metathesaurus (via the *hypernymy *and *other related *relations among concepts) and the Semantic Network (via the *associated with *relation among semantic types). Correct mappings would be strongly connected to other concepts while incorrect ones would be strongly disconnected since they are expected to be unrelated to the topic of the document. Consequently, correctly disambiguated concepts will be given a higher salience than the others in the concept clustering step, and their influence in the sentence selection step will be greater.

• Finally, one should stress the fact that the context unit for disambiguation used by the summarizer is the sentence in which the target ambiguous terms appear. In the experiments performed for evaluating WSD, the context used were MEDLINE citations, which provide more information for disambiguation than single sentences.

## Conclusions

This paper explores the effect of word ambiguity in biomedical summarization, and the correlation that exists between the accuracy of different WSD methods and the quality of the summaries that are generated when such methods are used as part of a summarization system for mapping documents onto concepts in the UMLS Metathesaurus. To this end, we compare the results achieved by three different WSD algorithms in the disambiguation task itself with those obtained when such algorithms are used as part of a graph-based summarizer.

We found that the use of WSD algorithms that choose among ambiguous MetaMap candidates in our UMLS-based approach to automatic summarization has a positive impact on the summarization results. However, this improvement is less than expected and is probably due to (1) errors made by the WSD systems (note that the best algorithm, AEC, presents an overall accuracy of 83.8%, but has been only evaluated over a relatively small set of ambiguous terms) and (2) the fact that, even when no explicit disambiguation is accomplished, the summarizer itself is implicitly performing some sort of WSD, as already discussed.

Besides, the improvement achieved in summarization by using WSD does not exhibit a direct relationship with the disambiguation performance. The most likely reason for this is that the errors in disambiguation are not equally important, but depend on the relative salience of the different concepts in the document to be summarized.

In spite of this, we have shown that, when both the WSD algorithms and the summarizer are assessed over large and homogeneous evaluation collections, there exists a correlation among the overall results of the WSD and summarization tasks, and the best WSD algorithm in the first task tends to be the best one in the second task. However, in order to generalize this finding to any summarization system, in the near future we plan to repeat the experiments presented in this work using other types of summarizers, for instance, a statistical summarizer.

## Authors' contributions

LP developed the method for automatic summarization and carried out the summarization evaluation experiments. AJ participated in the development of the WSD methods and carried out the WSD evaluation experiments. LP and AJ drafted the manuscript. AD and AA participated in the design of the experiments and reviewed the manuscript. All authors read, commented and approved the final version of the manuscript.
